# Geometric reconstruction of the left ventricle on a beating heart through a minimally invasive approach from the left anterolateral thoracotomy: case report

**DOI:** 10.3389/fcvm.2024.1507222

**Published:** 2024-12-04

**Authors:** R. N. Aigumov, S. A. Donakanyan, V. Yu Merzlyakov, A. I. Skopin, R. K. Baichurin, Z. G. Panagov, E. M. Sizhazhev, V. A. Shvartz, E. Z. Golukhova

**Affiliations:** Bakulev Scientific Center for Cardiovascular Surgery, Moscow, Russia

**Keywords:** left ventricle reconstruction, mini-invasive approach, cardiovascular pathology, ventricular aneurysm, cardiovascular research, case report

## Abstract

Despite the widespread use of mini-invasive treatment methods in cardiac surgery, their use in post-infarction myocardial aneurysms of the left ventricle is not of frequent occurrence. In this clinical case, we used left anterolateral thoracotomy and “eating heart” technique to correct the post-infarction left ventricle aneurysm with ventricular thrombosis using the Dor method in a 66-year-old patient. This technique created opportunity to perform safely and effective the planned reconstruction of the left ventricle with less trauma, as well as to ease the postoperative course and recovery of the patient, reduce hospitalization time.

## Introduction

Size enlargement, shape distortion, and dysfunction of the left ventricle (LV) due to necrosis and scarring are characteristic signs of postinfarction left ventricular aneurysm (PLVA). These pathological processes are the main target of surgical intervention, the purpose of which is to isolate devitalized tissues with restoration of the elliptical shape and proper dimensions of the LV (1). The Dor procedure is among the top recognized surgical techniques used for this purpose.

The standard Dor procedure is a geometric reconstruction of the LV performed through a median sternotomy under conditions of cardioplegic arrest (2).

The advancement of this technique has led to its implementation on a beating heart under cardiopulmonary bypass (CPB) (3–6).

The world literature includes just a few publications dedicated to the experience of performing LV reconstruction through left anterior minithoracotomy in a stopped heart (7–9).

In this report, we describe a case of a successful Dor procedure performed under conditions of minimal access and parallel perfusion.

## Case description

**Figure 1:**
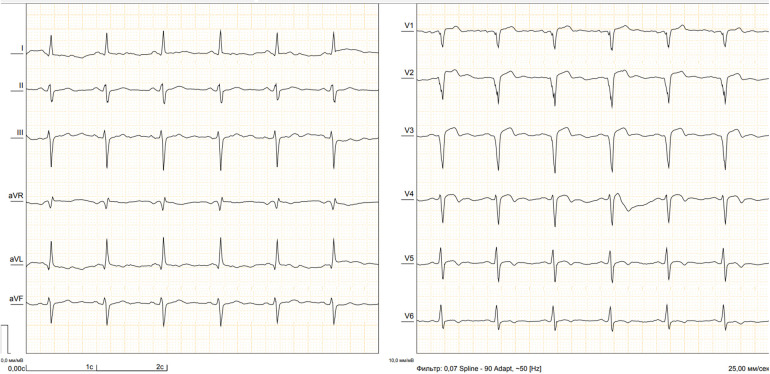
Electrocardiogram of the patient.

**Figure 2:**
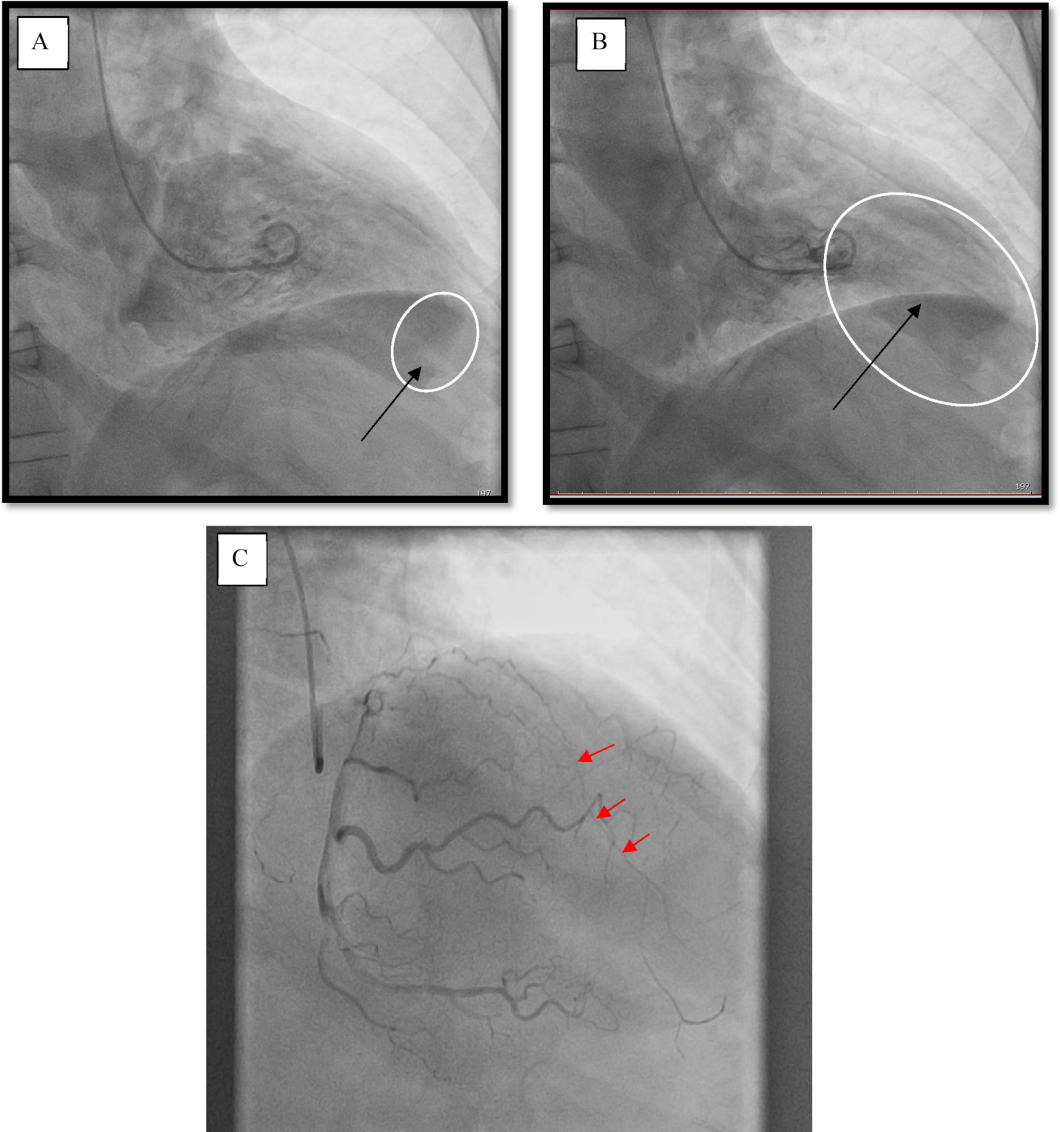
Left ventriculography. **(A)** Diastole, the arrow points at a thrombus at the left ventricle apex. **(B)** Systole, the arrow indicates the area of the aneurysm. **(C)** Coronary catheterization. The arrows point at the occlusion of the left anterior descending branch of the left coronary artery (LAD LCA).

A male patient (66 years of age, weighing 80 kg) was sent to our Center with signs of the functional class III heart failure and diagnosed with thrombosed PLVA. Upon admission to the department, the diagnosis was confirmed by electrocardiography, echocardiography (EchoCG), and ventriculography. The ECG is specific, it is shown in Figure 1. EchoCG data revealed an enlarged LV with the left ventricular end-diastolic volume (LV EDV) of 177 ml, end-sistolic volume (LV ESV)—116.8 ml, EDV index—88.5 ml/m^2^, ESV index—58.4 ml/m^2^, a change in its shape (left ventricular sphericity index of 0.65 in diastole and apical conicity index of 0.93 in systole), areas of hypo- and akinesia with the formation of a thrombosed aneurysm of anterolateral segment with transition to the apex (aneurysm volume of 56 ml, thrombus size of 17 × 29 mm), and also a reduction in global contractility of the LV myocardium characterized by the left ventricular ejection fraction (LVEF) of 34%. Left ventriculography in two projections confirmed the fact of pronounced negative remodeling of the LV in the form of an aneurysm with thrombus formation (Figures 2A,B).

According to the results of coronary catheterization, occlusion of the left anterior descending branch of the left coronary artery (LAD LCA) was noted without proper filling of the lumen along intersystem and intrasystem collaterals (Figure 2C).

It should be noted that this patient had a history of cerebral circulatory disorders (transient ischemic attack).

Considering the high functional class of chronic heart failure, the large volume of the aneurysm, LV dysfunction with LVEF under 35%, as well as the presence of a life-threatening thrombus in the left ventricular lumen, the only predictably effective treatment method was open-heart bypass surgery.

## Surgical intervention

**Figure 3:**
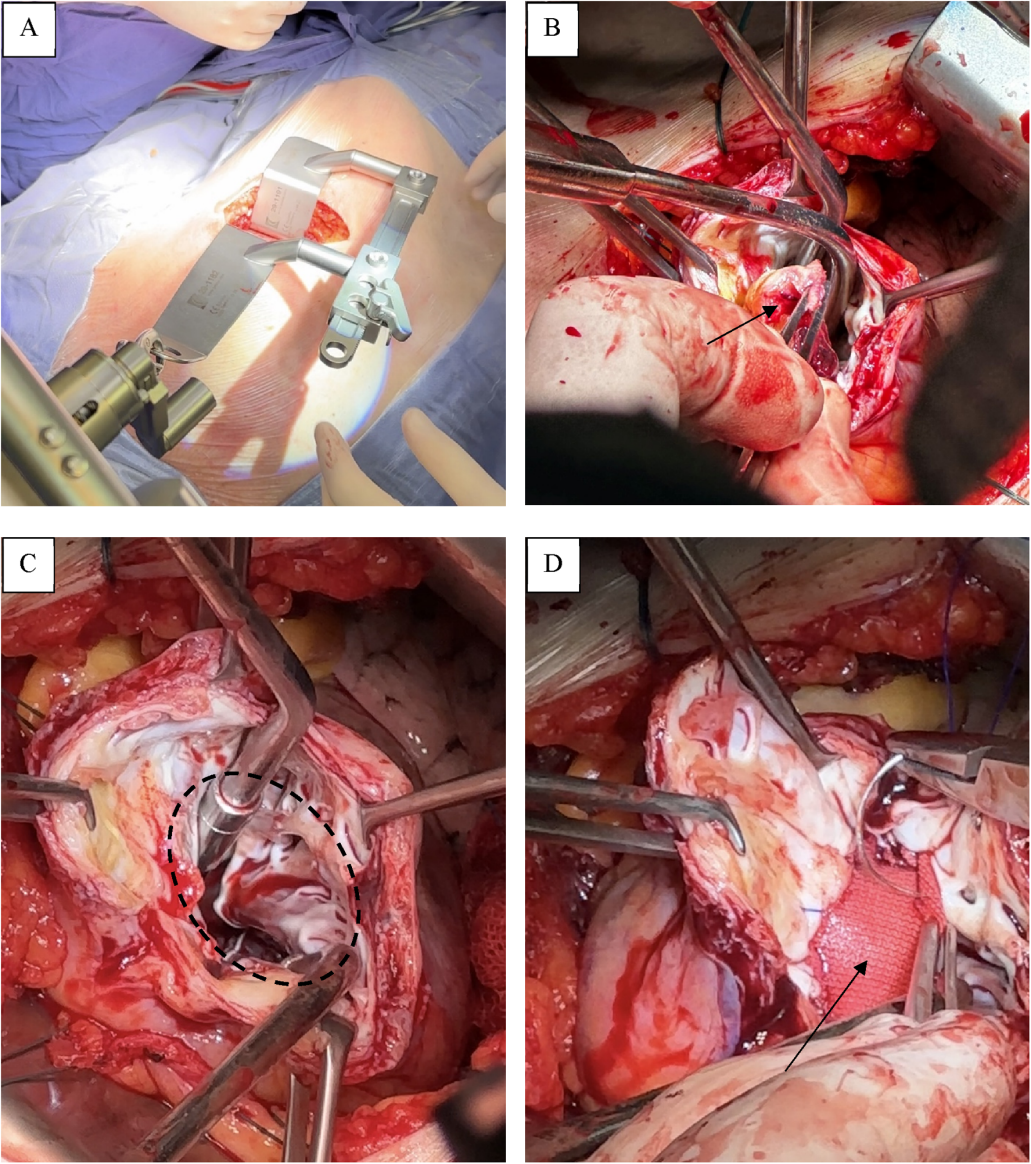
Operative stage. **(A)** A left anterolateral minithoracotomy in the fifth intercostal space. **(B)** Thrombus. **(C)** Boundaries of the viable myocardium. **(D)** Synthetic patch made of Dacron.

The heart was accessed through a left anterolateral minithoracotomy in the fifth intercostal space (Figure 3A). The surgery was performed under conditions of parallel perfusion with the connection of peripheral CPB through the left femoral vein and femoral artery. The venous cannula was installed in the right atrium closer to the superior vena cava under the control of transesophageal EchoCG. After opening the pericardium and its fixation, we performed a ventriculotomy, removed the thrombus (Figure 3B), and then, under full visualization of the boundaries of the viable myocardium (Figure 3C), we carried out a geometric reconstruction of the LV following the Dor procedure, using a synthetic patch made of Dacron (polyethylene terephthalate) (Figure 3D).

**Figure 4:**
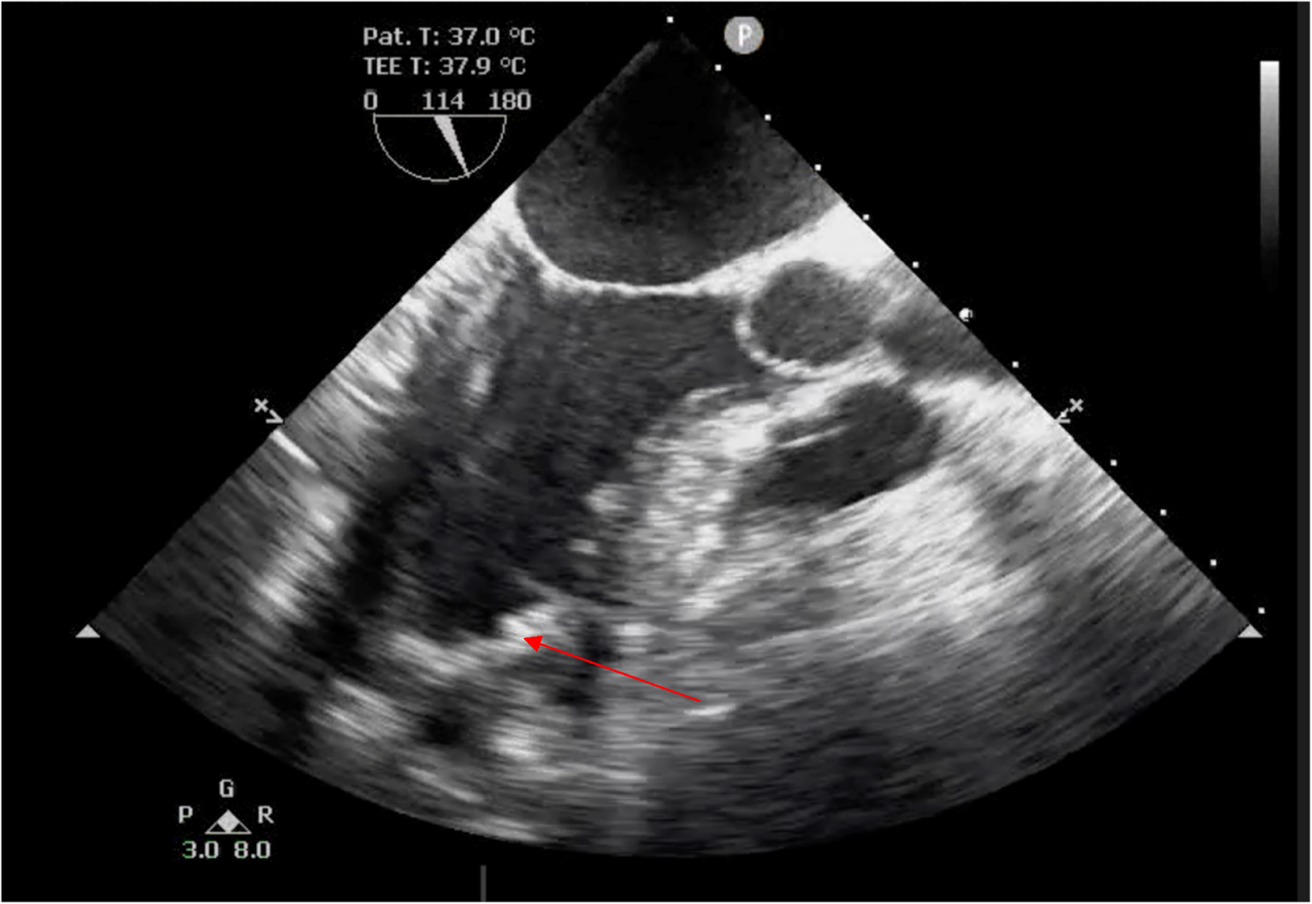
TEE after the surgery. LV, systole. The arrow points at the patch.

The duration of CPB was 36 min. The time of artificial lung ventilation was 11 h. There was no need for cardiotonic agents. The next day after the surgery, the patient was transferred to the general ward from the intensive care unit, and verticalization of the patient was attempted. He was discharged in stable condition on the fifth day. Basic EchoCG data at discharge were as follows: left ventricular end-systolic volume, LV EDV = 135.3 ml (EDV index—67.6 ml/m^2^), LV ESV = 62.0 ml (ESV index—31 ml/m^2^); LVEF = 54.2%. TEE after the surgery shows physiologically more correctly formed LV geometry (Figure 4).

## Discussion

This clinical case reports the possibility of adequately performing complex surgical reconstruction on the LV by means of a minithoracotomy and on a beating heart under CPB conditions.

The Dor procedure was first developed in the mid-1980s as a novel method for surgical treatment of PLVA. It involves endoventricular LV repair using a circumferential pursestring suture and a circular patch (2). We performed this complex intervention under standard conditions: median sternotomy and cardioplegic arrest of the heart (2). To date, this approach remains the gold standard worldwide in the surgery of LV aneurysms. However, over the past two decades, interest in minimally invasive procedures in cardiac surgery has increased, which has led to an expansion of the arsenal of surgical approaches, thereby eliminating traditional, more traumatic surgical conditions.

Considering possible complications associated with cardioplegic arrest of the heart, the method of surgical treatment of PLVA on a beating heart has become an effective alternative. Besides, it provides better visualization of the boundaries of the viable myocardium (3–6).

As part of achieving minimal invasiveness, the possibilities of using various surgical approaches instead of the more traumatic median sternotomy have expanded. In this case, the optimal mini-access is a left anterolateral thoracotomy.

The techniques of MIDCAB as a form of off-pump coronary artery bypass surgery and MICS CABG as an advanced minimally invasive technique for multi-vessel bypass, performed through this approach, demonstrate the option of performing technical actions with good results not only in the area of the apex and anterior wall of the heart, but virtually over its entire surface, which makes left anterior minithoracotomy highly attractive (10).

Previously, the possibility, effectiveness and safety of the LV surgical reconstruction through left anterior minithoracotomy was verified by colleagues from Europe, but all procedures were performed under conditions of cardioplegia and aortic cross-clamping through the port-access approach (7, 8) or additional thoracotomy (9).

Hence, our surgical strategy was to combine these two attractive minimally invasive approaches to the treatment of LV aneurysms.

LV thrombosis was also an important factor determining the indications for this operation, in addition to the high functional class of chronic heart failure and a large LV aneurysm, which occupied almost a third of the total LV volume with decreased global myocardial contractility. This factor was regarded as a life—threatening source of thromboembolic complications, taking into account patient's aggravating neurological history—a postponed episode of a cerebrovascular event.

During the preoperative planning of the intervention strategy, taking into account the need for only isolated treatment of LV aneurysm without CABG and the patient's preference, we chose the method of surgical intervention through a minimally invasive approach on a beating heart, which was a pioneering method in Russia. Furthermore, we did not find any publications in the worldwide reference databases dedicated to the experience of performing such operations.

The condition in which the venous cannula reaches the lumen of the superior vena cava, as well as vacuum-assisted venous drainage, are very important, because this allows for maximum LV unloading and, consequently, provides optimal and safe visualization.

Performing surgery on a beating heart made it possible to more clearly visualize the demarcation zone and properly isolate the scarred part of the aneurysm.

It is necessary to keep the patient's head slightly lowered and keep sufficient perfusion pressure to prevent the air embolism during the main stage of the operation. After careful removal of the thrombus, a thorough examination of the LV cavity for the presence of foreign bodies was performed. Further, enough blood is released from this cavity under pressure to completely evacuate possible hidden emboli. The presence of aortic insufficiency is a contraindication for performing this procedure. The final sutures on the patch are applied in conditions of a filled heart after complete deaeration through the left hole, then sealing with auto-tissues is achieved.

The technique of a “Beating heart” allows minimizing the risks of developing of the small cardiac output syndrome associated with an excessive decrease of the LV cavity volume, since the boundary between a viable and non-viable myocardium is clearly visualized intraoperatively. This boundary is a guideline for preoperative intervention design based on echocardiography, ventriculography/MRI data. Such a concept makes it possible to achieve not only the optimal volume, but also the shape of the LV (11).

The outcome of this surgery is impressive. This method provides an early mobilization of the patient (already on the first day after surgery) and his or her early discharge (on the fourth or fifth day). Values of indicators obtained with a control EchoCG imply proper isolation of the affected area, while maintaining optimal LV volume. There were no signs of heart failure in the postoperative period and, accordingly, there was no need for infusion of cardiotonic preparations, which is one of the advantages of the intervention without cardioplegia.

The skills of minimally invasive CABG, as well as the accumulation of experience in minimally invasive LV reconstruction on a beating heart, will expand the potential of combined intervention for complex coronary artery disease in the future.

Further studies using larger sample sizes and long-term outcome studies are needed.

## Conclusion

Left anterolateral minithoracotomy is an approach that allows proper surgical reconstruction of the LV on a beating heart with good results. This approach provides optimal surgical field visibility and can be used in patients with PLVAs. Future large-scale studies analyzing long-term results will allow researchers to draw unambiguous conclusions.

## Data Availability

The original contributions presented in the study are included in the article/Supplementary Material, further inquiries can be directed to the corresponding author.
